# Supplementary material for: Modelling Skylarks (Alauda arvensis) to Predict Impacts of Changes in Land Management and Policy: Development and Testing of an Agent-Based Model
Source: PLoS One. 2013 Jun 6;8(6):e65803. doi: 10.1371/journal.pone.0065803 (PMC3675089; doi:10.1371/journal.pone.0065803)
Supplement: Supporting Information S4 — The skylark ODdox as a zipped archive. (ZIP) [file pone.0065803.s004.zip › Skylark_ODdox/class_skylark___pre_fledgeling.html]

ALMaSS Skylark ODdox: Skylark\_PreFledgeling Class Reference


ALMaSS Skylark ODdox
 2.0


- Main Page
- Related Pages
- Classes
- Files

- Class List
- Class Index
- Class Hierarchy
- Class Members

Public Member Functions |
Protected Member Functions

Skylark\_PreFledgeling Class Reference

`#include <skylarks_all.h>`

List of all members.

|  |  |
| --- | --- |
| Public Member Functions | |
| virtual void | BeginStep (void) |
| virtual void | EndStep (void) |
|  | Skylark\_PreFledgeling (int x, int y, Landscape \*L, SkTerritories \*Terrs, Skylark\_Male \*Daddy, bool sex, double size, int age, Skylark\_Population\_Manager \*SPM, int bx, int by, int mh) |
| virtual void | Step (void) |
| Public Member Functions inherited from Skylark\_Nestling | |
| virtual double | On\_FoodSupply (double food) |
| void | OnDadDead () |
| void | OnDeserted () |
| void | OnYouHaveBeenEaten () |
|  | Skylark\_Nestling (int x, int y, Skylark\_Male \*Daddy, Landscape \*L, SkTerritories \*Terrs, Skylark\_Population\_Manager \*SPM, int bx, int by, int mh) |
| Public Member Functions inherited from Skylark\_Base | |
| void | AddStriglingMort (int lifestage) |
| bool | InSquare (rectangle R) |
|  | Skylark\_Base (int x, int y, SkTerritories \*Terrs, Landscape \*L, Skylark\_Population\_Manager \*SPM, int bx, int by, int mh) |
| virtual int | WhatState () |
| Public Member Functions inherited from TAnimal | |
| void | CheckManagement (void) |
| void | CheckManagementXY (int x, int y) |
| virtual void | CopyMyself () |
| virtual void | Dying () |
| virtual void | KillThis () |
| void | SetX (int a\_x) |
| void | SetY (int a\_y) |
| int | Supply\_m\_Location\_x () |
| int | Supply\_m\_Location\_y () |
| unsigned | SupplyFarmOwnerRef () |
| int | SupplyPolygonRef () |
| AnimalPosition | SupplyPosition () |
|  | TAnimal (int x, int y, Landscape \*L) |
| Public Member Functions inherited from TALMaSSObject | |
| void | OnArrayBoundsError () |
|  | TALMaSSObject () |
| virtual | ~TALMaSSObject () |

|  |  |
| --- | --- |
| Protected Member Functions | |
| double | GetFledgelingEM (int Age) |
| double | GetFood () |
| virtual bool | OnFarmEvent (FarmToDo event) |
| virtual void | PesticideResponse () |
| virtual int | st\_Developing () |
| virtual void | st\_Dying () |
| virtual void | st\_Maturing () |

|  |  |
| --- | --- |
| Additional Inherited Members | |
| Protected Attributes inherited from Skylark\_Nestling | |
| Skylark\_Male \* | m\_Dad |
| double | m\_EM |
| int | m\_EM\_fail |
| double | m\_GrNeed |
| int | m\_NestLeavingChance |
| bool | Sex |

---

## Constructor & Destructor Documentation

|  |  |  |  |
| --- | --- | --- | --- |
| Skylark\_PreFledgeling::Skylark\_PreFledgeling | ( | int | *x*, |
|  |  | int | *y*, |
|  |  | Landscape \* | *L*, |
|  |  | SkTerritories \* | *Terrs*, |
|  |  | Skylark\_Male \* | *Daddy*, |
|  |  | bool | *sex*, |
|  |  | double | *size*, |
|  |  | int | *age*, |
|  |  | Skylark\_Population\_Manager \* | *SPM*, |
|  |  | int | *bx*, |
|  |  | int | *by*, |
|  |  | int | *mh* |
|  | ) |  |  |

References Skylark\_Base::Age, Skylark\_Base::m\_CurrentSkState, Skylark\_Nestling::m\_EM, Skylark\_Nestling::m\_GrNeed, Skylark\_Base::m\_Size, Skylark\_Nestling::Sex, and toss\_PDeveloping.

:

Skylark\_Nestling( x, y, Daddy, L, Terrs, SPM, bx, by, mh ) {

Sex = sex; // must be reset because the nestling constructor can change it

m\_Size = size;

Age = age;

m\_EM = 0;

m\_GrNeed = 0;

m\_CurrentSkState = toss\_PDeveloping; // must develop today

}

---

## Member Function Documentation

|  |  |  |  |  |  |
| --- | --- | --- | --- | --- | --- |
| void Skylark\_PreFledgeling::BeginStep | ( | void |  | ) |  |

virtual

Reimplemented from Skylark\_Nestling.

References TAnimal::CheckManagement().

{

CheckManagement();

}

|  |  |  |  |  |  |
| --- | --- | --- | --- | --- | --- |
| void Skylark\_PreFledgeling::EndStep | ( | void |  | ) |  |

virtual

Reimplemented from Skylark\_Nestling.

References TALMaSSObject::CurrentStateNo, Calendar::Date(), g\_date, Skylark\_Base::m\_CurrentSkState, PesticideResponse(), st\_Developing(), st\_Dying(), st\_Maturing(), TALMaSSObject::StepDone, toss\_Destroy, toss\_PDeveloping, toss\_PDying, toss\_PMaturing, and Landscape::Warn().

{

#ifdef \_\_CJTDebug\_5

if ( IsAlive() != 0x0DEADC0DE ) DEADCODEError();

#endif

if ( m\_CurrentSkState == toss\_Destroy ) return;

#ifdef \_\_CJTDebug\_5

if ( Dad )

if ( !Dad->DoIExistP( this ) ) {

g\_land->Warn( "Skylark\_PreFeldgeling::EndStep(): DeBug5 ", NULL );

exit( 1 );

}

#endif

switch ( m\_CurrentSkState ) {

case toss\_PDeveloping: // Develop

switch ( st\_Developing() ) {

case 2:

#ifdef TEST\_ISSUE\_DEATH\_WARRANT

printf( "Skylark\_PreFledgeling::EndStep() : st\_Developing() : %d\n", ( int )g\_date->Date() );

#endif

m\_CurrentSkState = toss\_PDying; // -> die

break;

case 1:

m\_CurrentSkState = toss\_PMaturing; // -> Maturing

// This state must be completed now so make the call from here to stop

// it being made the next day

st\_Maturing();

m\_CurrentSkState = toss\_Destroy; // will kill it at end of step!

CurrentStateNo = -1;

StepDone = true;

break;

}

break;

case 8: // Maturing

g\_land->Warn( "Skylark\_PreFledling::EndStep(): Maturing ", NULL );

exit( 1 );

case 9: // Dying

st\_Dying();

StepDone = true;

break;

default:

/\* char errornum[20]; sprintf(errornum, "%d", m\_CurrentSkState );

g\_land->Warn("Skylark\_PreFledgeling::EndStep(): Unknown state: ", errornum); exit(0); \*/

break;

}

// Need to find out if it is poisoned

#ifdef \_\_PESTICIDE\_RA

PesticideResponse();

#endif

}

|  |  |  |  |  |  |
| --- | --- | --- | --- | --- | --- |
| double Skylark\_PreFledgeling::GetFledgelingEM | ( | int | *Age* | ) |  |

protected

References Skylark\_Base::m\_OurPopulationManager, and Skylark\_Population\_Manager::SupplyEMi().

{

#ifdef \_\_CJTDebug\_5

if ( IsAlive() != 0xDEADC0DE )

DEADCODEError();

#endif

// Speed optimisation - this is the same for every bird so done in PopMan

return m\_OurPopulationManager->SupplyEMi(); // in gdw needed

}

|  |  |  |  |  |
| --- | --- | --- | --- | --- |
| double Skylark\_PreFledgeling::GetFood | ( |  | ) |  |

protected

References Skylark\_Base::Age, Skylark\_Base::m\_OurPopulationManager, and Skylark\_Population\_Manager::SupplyEMi().

{

#ifdef \_\_CJTDebug\_5

if ( IsAlive() != 0x0DEADC0DE ) DEADCODEError();

#endif

// Food it obtained from the local area

// This will depend upon the habitat quality/weather & searching efficiency

// This is possibly too complicated just now so:

// First prototype model will assume that for the last 10 days the birds

// can find 10% of their need for each day after 20 days so at 30 days they

// are completely independent and cannot starve

// 1st prototype

double food;

if (Age>=20) {

food = m\_OurPopulationManager->SupplyEMi() \* 0.1 \* ( Age - 20 );

} else food=0;

return food;

}

|  |  |  |  |  |  |
| --- | --- | --- | --- | --- | --- |
| bool Skylark\_PreFledgeling::OnFarmEvent | ( | FarmToDo | *event* | ) |  |

protectedvirtual

Reimplemented from Skylark\_Nestling.

References Skylark\_Base::AddStriglingMort(), Skylark\_Base::Age, autumn\_harrow, autumn\_or\_spring\_plough, autumn\_plough, autumn\_roll, autumn\_sow, burn\_straw\_stubble, cattle\_out, cattle\_out\_low, cut\_to\_hay, cut\_to\_silage, cut\_weeds, Calendar::Date(), deep\_ploughing, Landscape::EventtypeToString(), fa\_ammoniumsulphate, fa\_greenmanure, fa\_manure, fa\_npk, fa\_pk, fa\_sludge, fa\_slurry, fp\_greenmanure, fp\_liquidNH3, fp\_manganesesulphate, fp\_manure, fp\_npk, fp\_npks, fp\_pk, fp\_sludge, fp\_slurry, fungicide\_treat, g\_date, glyphosate, growth\_regulator, harvest, hay\_bailing, hay\_turning, herbicide\_treat, hilling\_up, insecticide\_treat, Skylark\_Base::m\_CurrentSkState, TAnimal::m\_OurLandscape, molluscicide, mow, pigs\_out, product\_treat, random(), row\_cultivation, sleep\_all\_day, spring\_harrow, spring\_plough, spring\_roll, spring\_sow, straw\_chopping, strigling, strigling\_sow, stubble\_harrowing, swathing, syninsecticide\_treat, toss\_PDying, trial\_insecticidetreat, CfgInt::value(), Landscape::Warn(), water, and winter\_plough.

{

switch ( event ) {

case sleep\_all\_day:

case fp\_npks:

case fp\_npk:

case fp\_pk:

case fp\_slurry:

case fp\_manganesesulphate:

case fp\_manure:

case fp\_greenmanure:

case fp\_sludge:

case fa\_npk:

case fa\_pk:

case fa\_slurry:

case fa\_ammoniumsulphate:

case fa\_manure:

case fa\_greenmanure:

case fa\_sludge:

case herbicide\_treat:

case growth\_regulator:

case fungicide\_treat:

case insecticide\_treat:

case trial\_insecticidetreat:

case molluscicide:

case row\_cultivation:

case water:

case swathing:

case harvest:

case cattle\_out:

case cattle\_out\_low:

case hay\_bailing:

case mow:

case cut\_weeds:

case pigs\_out:

break;

case autumn\_harrow:

case autumn\_roll:

case autumn\_sow:

case winter\_plough:

case deep\_ploughing:

case spring\_plough:

case spring\_harrow:

case spring\_roll:

case spring\_sow:

case autumn\_plough:

case hilling\_up:

case cut\_to\_hay:

case cut\_to\_silage:

case straw\_chopping:

case hay\_turning:

case stubble\_harrowing:

case autumn\_or\_spring\_plough:

case burn\_straw\_stubble:

if ( Age <= 20 ) {

#ifdef TEST\_ISSUE\_DEATH\_WARRANT

printf( "Skylark\_PreFledgeling::OnFarmEvent() : %d : %d\n", event, ( int )g\_date->Date() );

#endif

m\_CurrentSkState = toss\_PDying;

}

break;

case fp\_liquidNH3:

break;

case syninsecticide\_treat:

if ( random( 1000 ) < cfg\_insecticide\_direct\_mortP.value() ) {

#ifdef TEST\_ISSUE\_DEATH\_WARRANT

printf( "Skylark\_PreFledgeling::OnFarmEvent() : %d : %d\n", event, ( int )g\_date->Date() );

#endif

m\_CurrentSkState = toss\_PDying;

}

break;

case strigling\_sow:

case strigling:

#ifndef \_\_NoStriglingEffect

if ( ( Age <= 20 ) && ( random( 100 ) < 50 ) ) {

#ifdef TEST\_ISSUE\_DEATH\_WARRANT

printf( "Skylark\_PreFledgeling::OnFarmEvent() : %d : %d\n", event, ( int )g\_date->Date() );

#endif

m\_CurrentSkState = toss\_PDying;

AddStriglingMort( 3 );

}

#endif

break;

case product\_treat:

break;

case glyphosate:

break;

default:

g\_land->Warn( "Skylark\_PreFledgeling::OnFarmEvent(): ""Unknown event type:",

m\_OurLandscape->EventtypeToString( event ) );

exit( 1 );

}

if ( m\_CurrentSkState == toss\_PDying ) return true; else

return false;

}

|  |  |  |  |  |
| --- | --- | --- | --- | --- |
| virtual void Skylark\_PreFledgeling::PesticideResponse | ( |  | ) |  |

protectedvirtual

Reimplemented from Skylark\_Nestling.

Referenced by EndStep().

|  |  |  |  |  |
| --- | --- | --- | --- | --- |
| int Skylark\_PreFledgeling::st\_Developing | ( |  | ) |  |

protectedvirtual

Reimplemented from Skylark\_Nestling.

References Skylark\_Base::Age, Skylark\_Base::DailyMortality(), Skylark\_Nestling::m\_EM, Skylark\_Nestling::m\_GrNeed, Skylark\_Base::m\_Size, and PreFledgeMortProb.

Referenced by EndStep().

{

#ifdef \_\_CJTDebug\_5

if ( IsAlive() != 0x0DEADC0DE ) DEADCODEError();

#endif

// Timing: called at the end of each day

if ( DailyMortality( PreFledgeMortProb ) ) {

//m\_OurPopulationManager->WriteToTest2File(m\_OurLandscape->SupplyDayInYear(),3001);

return 2; //transfer to state die

}

if ( Age++ == 31 ) {

//m\_OurPopulationManager->WriteToTest2File((int)3000, 3000);

m\_Size = 38.0; // Give it a size

return 1; // Next stage

} else {

// Get Next days EM

m\_EM = 0; // m\_OurPopulationManager->SupplyEMi(); // KCal

m\_GrNeed = 0; // gdw

return 0; // still developing

}

}

|  |  |  |  |  |
| --- | --- | --- | --- | --- |
| void Skylark\_PreFledgeling::st\_Dying | ( |  | ) |  |

protectedvirtual

Reimplemented from Skylark\_Nestling.

References TALMaSSObject::CurrentStateNo, Skylark\_Base::m\_CurrentSkState, Skylark\_Nestling::m\_Dad, Skylark\_Male::OnPreFledgelingDeath(), and toss\_Destroy.

Referenced by EndStep().

{

#ifdef \_\_CJTDebug\_5

if ( IsAlive() != 0x0DEADC0DE ) DEADCODEError();

#endif

if ( m\_Dad != NULL ) m\_Dad->OnPreFledgelingDeath( this );

m\_CurrentSkState = toss\_Destroy; // will kill it at end of step!

CurrentStateNo = -1;

}

|  |  |  |  |  |
| --- | --- | --- | --- | --- |
| void Skylark\_PreFledgeling::st\_Maturing | ( |  | ) |  |

protectedvirtual

Reimplemented from Skylark\_Nestling.

References AdultSkylark\_struct::age, Skylark\_struct::bx, Skylark\_struct::by, Skylark\_Population\_Manager::CreateObjects(), Skylark\_struct::L, Skylark\_Base::m\_Born\_x, Skylark\_Base::m\_Born\_y, Skylark\_Nestling::m\_Dad, TAnimal::m\_Location\_x, TAnimal::m\_Location\_y, Skylark\_Base::m\_MyHome, TAnimal::m\_OurLandscape, Skylark\_Base::m\_OurPopulationManager, Skylark\_Base::m\_OurTerritories, Skylark\_Base::m\_Size, Skylark\_struct::mh, Skylark\_Male::OnPreFledgelingMature(), AdultSkylark\_struct::sex, Skylark\_Nestling::Sex, AdultSkylark\_struct::size, Skylark\_struct::Terrs, Skylark\_struct::x, and Skylark\_struct::y.

Referenced by EndStep().

{

#ifdef \_\_CJTDebug\_5

if ( IsAlive() != 0x0DEADC0DE ) DEADCODEError();

#endif

// Timing: occurs at the end of the day

// Mortality after this time, until they return is taken up in the

// immigration mortality (total 35%)

AdultSkylark\_struct \* aps;

aps = new AdultSkylark\_struct;

aps->x = m\_Location\_x;

aps->y = m\_Location\_y;

aps->size = m\_Size;

aps->Terrs = m\_OurTerritories;

aps->L = m\_OurLandscape;

aps->sex = Sex;

aps->age = 0;

aps->bx = m\_Born\_x;

aps->mh = m\_MyHome;

aps->by = m\_Born\_y;

if ( Sex == true ) m\_OurPopulationManager->CreateObjects( 3, this, NULL, aps, 1 ); else

m\_OurPopulationManager->CreateObjects( 4, this, NULL, aps, 1 );

// object will be destroyed by death state

// but must let Dad know anyway

delete aps;

if ( m\_Dad != NULL ) m\_Dad->OnPreFledgelingMature( this );

// m\_OurPopulationManager->WriteToTest2File(3,3);

}

|  |  |  |  |  |  |
| --- | --- | --- | --- | --- | --- |
| void Skylark\_PreFledgeling::Step | ( | void |  | ) |  |

virtual

Reimplemented from Skylark\_Nestling.

References Skylark\_Base::m\_CurrentSkState, TALMaSSObject::StepDone, toss\_Destroy, toss\_Initiation, toss\_PDeveloping, toss\_PDying, toss\_PMaturing, and Landscape::Warn().

{

#ifdef \_\_CJTDebug\_5

if ( IsAlive() != 0x0DEADC0DE ) DEADCODEError();

#endif

if ( StepDone ) return;

switch ( m\_CurrentSkState ) {

case toss\_Initiation: // Initial

// Should never access this code becuase it should go

// to m\_CurrentSkState=toss\_PDeveloping;

g\_land->Warn( "Skylark\_PreFeldgeling::Step(): Ilegal ", NULL );

exit( 1 );

case toss\_PDeveloping: // Develop

StepDone = true;

break;

case toss\_PMaturing: // Maturing

StepDone = true;

break;

case toss\_PDying: // Dying

StepDone = true;

break;

case toss\_Destroy:

StepDone = true;

break;

default:

g\_land->Warn( "Skylark\_PreFeldgeling::Step(): Unknown State ", NULL ); exit( 1 );

}

}

---

The documentation for this class was generated from the following files:

- skylarks\_all.h
- skylarks\_all.cpp


- Skylark\_PreFledgeling
- Generated on Thu Jan 10 2013 13:15:38 for ALMaSS Skylark ODdox by
   1.8.1.1
